# Editorial

**Published:** 1851-02

**Authors:** 


### THE MEDICAL EXAMINER.

PHILADELPHIA, FEBRUARY, 1851.

### AMERICAN MEDICAL ASSOCIATION.

The Committee of Arrangements request all Societies and Medical Institutions authorized to send delegates, to forward a correct list of those selected to attend the next annual meeting, to the Secretary, Dr. H. W. De Saussure, at Charleston, S. C., on, or before the first day of April.

In consequence of the resignation of Dr. Stillé, one of the Secretaries, from ill health, all communications intended for the next meeting of the Association, must be addressed to the remaining Secretary, Dr. H. W. De Saussure, Charleston S. C.

The fourth Annual Meeting of the American Medical Association, will be held at Charleston, S. C., on the second Tuesday of May next.—*Charleston Med. Jour. and Review.*

---

RANKING'S ABSTRACT FOR JANUARY, 1851.

We have received the English copy of the above work simultaneously with the American reprint, which the publishers, Messrs. Lindsay & Blakiston, have laid before their subscribers with most commendable dispatch, notwithstanding the large number of illustrations which accompany it.

---

THE STETHOSCOPE AND VIRGINIA MEDICAL GAZETTE.

We welcome, with great pleasure, the appearance of a new Medical Journal, under the above title, in Richmond, Va., edited by Dr. P. Claiborne Gooch. From the spirit and ability which are displayed in the first number, we anticipate an interesting addition to our list of exchanges, as well as a very agreeable organ of communication with many valued friends in the Profession of Virginia.

---

THE PHILADELPHIA LANCET,

Is the name of a new bi-monthly which has just been issued, and the first two numbers of which we have received. It seems to be conducted with spirit, and the editor declares his determination to wage war upon quackery in all its shapes, to oppose all reverence for men and cliques, and to furnish to his readers a condensed account of all that is new and valuable in the profession. We wish him all success.

---

The *St. Louis Probe* has ceased to exist. The editors have discovered that there is neither money nor fame to be gained in conducting a medical monthly. The Probe speaks *feelingly.*

---

TO A NEIGHBORING CORRESPONDENT.

We had received, and placed on file for insertion, a communication from a neighboring correspondent; but as he has sent it to another journal, which has published it, of course it cannot appear in this. Some of our readers, perhaps, are not aware of the understanding in these matters, that no communication should be sent to more than one journal, unless the first should decline to insert it.

---

SYDENHAM SOCIETY OF LONDON.

We are requested to state that it is no longer practicable to supply the books of the *first, second* and *third* years issued by the Sydenham Society.

### RICORD'S ILLUSTRATIONS OF VENEREAL DISEASE.

We have received from the publisher, Mr. A. Hart, a copy of the admirable Illustrations of Syphilitic disease, by P. Ricord, D. M. P., translated by Dr. T. F. Betton, and arranged by Dr. P. B. Goddard. We regret that the crowded state of our pages prevents us from giving, at present, more than this meagre announcement of the issue of this valuable work. At a future day we hope to do it justice. In the meantime, we can only say that these "illustrations" exceed anything of the kind that has ever appeared in this country as regards fidelity and beauty of execution.

---

The grand jury acting for the city and county of Philadelphia, have found a true bill of involuntary manslaughter, against Messrs. Chamberlain and McFadden, a druggist and his assistant, of this city, for causing the death of a patient by the administration of Sulph. of Morphia, instead of Sulphate of Quinine.

Although the lesson will be a bitter one to those involved, we trust it will not be without good results to many others who are grossly careless in compounding the prescriptions of physicians. In this case the prescription was written in a clear and legible hand, and the assistant on being asked to show what he had put up, produced the bottle containing *Sulphate of Morphia !*

---

### HONORS TO MEDICAL MEN IN ENGLAND.

The honor of the order of *Bath* has been conferred on the following named medical gentlemen, in testimonial of their distinguished services: Sir W. Burnett, M. D.; Sir James McGrigor, M. D.; James Thompson, Esq., to be Knights Commanders of Bath. The following "*Companions of the order of Bath:*" Henry Franklin, Esq.; Jas. French, M. D.; Sir J. R. Grant, M. D.; J. Gunning, Esq.; R. P. Hyllyar, Esq.; J. R. Hume, M. D.; Sir J. Liddell, M. D.; D. McArthur, M. D.; B. W. Macleod, M. D.; Sir G. Magrath, M. D.; B. F. Outram, M. D.; C. Renny, Esq.; Sir J. Richardson, M. D.; C. D. Straker, M. D.; Sir J. Webb; S. Woolriche, Esq.; J. Wylie, M. D.

The honor of Knighthood has recently been conferred on three distinguished British physicians; Dr. (now Sir) Robert Carswell, Physician to the King of the Belgians; Dr. (now Sir) Charles Hastings, the founder and late President of the Provincial Medical and Surgical Association; and Dr. (now Sir) B. F. Outram, C. B., Retired Inspector of Hospitals and Fleets.

We would like to see some share of the honors that are so freely lavished upon the officers of the Line, fall to the lot of the Medical officers in both our own services. In the Army, assimilated rank has been conferred upon the Medical Staff by an act of Congress, but in the Navy, they are battling still for the mere outward show of rank which the Line would fain deprive them of. If the services of gentlemen of education and refinement are desirable, (and in this respect our medical officers will compare with any in the world,) let them at least receive the courtesies due to them. They ask no more.

---

### Medical News.

The French Codex.—The Academy of Medicine is about to decide on the new remedies, which have been found useful, with the view to assign to such as are entitled to it, a place in the Pharmacopœia. It should be noticed, that no secret remedies can be sold in France, unless the formula has been submitted to the Minister of Commerce and Agriculture, who generally refers the matter to the Academy of Medicine, whose decision is invariably followed.

A good hit at Homœopathy.—Professor J. K. Mitchell, in his late Introductory, disposes of Homœopathy thus wittily and aptly:—A Physician educated in Philadelphia, says the Doctor, but who lapsed through a proclivous nature into Homœopathy, came to me once for a motto for a book which he was preparing on Homœopathy. As he usually consulted me when himself sick, and yet gave his microscopic doses to others, I felt no reluctance to give him a blow; so I said that once when a ranting lover on the stage cried out to his mistress, "My wound is great because 'tis small"—the witty Duke of Buckingham added from a side box—" Then 't had been greater were it none at all." Now, Doctor, said I, you can put that together thus—

> " My Physic's great because 'tis small,
> And would be greater were it none at all."

That is the whole argument of homœopathy in a couplet.

Death of a Distinguished Physician.—Dr. Royer Collard, Professor of Hygiene, at the Faculty of Medicine at Paris, died in that city in December last, at the early age of forty-seven.

John. S. Cameron, M. D., assistant Physician of the Marine Hospital, Staten Island, has fallen a victim to the typhus fever which prevails to a great extent among the newly arrived emigrants there.

